# Influences of Absorbed Dose Rate on the Mechanical Properties and Fiber–Matrix Interaction of High-Density Polyethylene-Based Carbon Fiber Reinforced Thermoplastic Irradiated by Electron-Beam

**DOI:** 10.3390/polym12123012

**Published:** 2020-12-16

**Authors:** Se Kye Park, Dong Yun Choi, Du Young Choi, Dong Yun Lee, Seung Hwa Yoo

**Affiliations:** 1 Daegyeong Division, Korea Institute of Industrial Technology, Yeongcheon 38822, Gyeongsangbuk-do, Korea; sekye@kitech.re.kr (S.K.P.); dychoi311@kitech.re.kr (D.Y.C.); 2 Department of Polymer Science and Engineering, Kyungpook National University, Daegu 41566, Gyeongsangbuk-do, Korea; dongyunlee@knu.ac.kr; 3 Carbon Materials Application R&D Group, Korea Institute of Industrial Technology, Jeonju 54853, Jeollabuk-do, Korea; duychoi@kitech.re.kr; 4 Department of Quantum System Engineering, College of Engineering, Jeonbuk National University, Jeonju 54896, Jeollabuk-do, Korea

**Keywords:** electron-beam irradiation, absorbed dose rate, carbon fiber-reinforced thermoplastic, tensile strength, fiber–matrix interaction

## Abstract

In this study, a high-density polyethylene (HDPE)-based carbon fiber-reinforced thermoplastic (CFRTP) was irradiated by an electron-beam. To assess the absorbed dose rate influence on its mechanical properties, the beam energy and absorbed dose were fixed, while the absorbed dose rates were varied. The tensile strength (TS) and Young’s modulus (YM) were evaluated. The irradiated CFRTP TS increased at absorbed dose rates of up to 6.8 kGy/s and decreased at higher rates. YM showed no meaningful differences. For CFRTPs constituents, the carbon fiber (CF) TS gradually increased, while the HDPE TS decreased slightly as the absorbed dose rates increased. The OH intermolecular bond was strongly developed in irradiated CFRTP at low absorbed dose rates and gradually declined when increasing those rates. X-ray photoelectron spectroscopy analysis revealed that the oxygen content of irradiated CFRTPs decreased with increasing absorbed dose rate due to the shorter irradiation time at higher dose rates. In conclusion, from the TS viewpoint, opposite effects occurred when increasing the absorbed dose rate: a favorable increase in CF TS and adverse decline of attractive hydrogen bonding interactions between HDPE and CF for CFRTPs TS. Therefore, the irradiated CFRTP TS was maximized at an optimum absorbed dose rate of 6.8 kGy/s.

## 1. Introduction

Fiber-reinforced plastics possess low specific density but are high-strength compared with metals; therefore, they are widely used in various industrial fields such as the automotive, aerospace, and medical industries [1,2,3,4]. Carbon fiber-reinforced plastics, in particular, have gained great interest, because they can effectively reduce the weight of composite materials by utilizing carbon fibers (CFs) that have 40 times higher specific strength than steel [5,6]. Intense research and development activities have been conducted recently on carbon fiber-reinforced thermoplastics (CFRTPs), owing to their advantages of high productivity and recyclability, because thermoplastics serve as matrix materials [7,8]. However, achieving satisfactory mechanical properties for CFRTPs has been a challenging task, because the low surface energy of CF results in poor interfacial adhesion with the thermoplastic matrix [9,10]. Therefore, several efforts have been made to overcome this limitation by functionalizing the CF surface using chemical (acid treatment, adding coupling agents) or physical (plasma, UV-ozone, ionizing radiation) treatments to improve the interfacial adhesion between CF and the thermoplastic matrix [11,12,13,14].

Chemical treatments of CF have several disadvantages compared to physical ones such as environmental pollution by inevitable use of toxic chemical agents, and the potential of deterioration in CFRTP mechanical properties when CF treatment is not conducted under precise control. In contrast to these chemical treatments, electron-beam irradiation technology is a rapid, simple, eco-friendly, and mass-producible processing method. Compared with other types of ionizing radiation, such as gamma rays or X-rays, electron-beams have a higher absorbed dose rate, which is advantageous for rapid processes and mass production [15,16]. Several works have studied pre-treatments using electron-beam irradiation for CF functionalization [17,18,19]. Our previous work attempted to irradiate HDPE-based CFRTPs by an electron-beam to enhance their mechanical properties [20]. Generally, with HDPE, it is difficult to obtain satisfactory mechanical properties of HDPE-based CFRTP because HDPE has poor flow properties, which limits infiltration into the spaces between fibers, and has poor interfacial interaction with CF due to the non-polarity of HDPE [21,22]. However, we found that the tensile strength of the irradiated HDPE-based CFRTP was enhanced by simply increasing the absorbed dose. Our results indicated that electron-beam irradiation induced an enhancement in interfacial interaction between CF and the HDPE matrix due to the formation of various polar oxygen-containing functional groups along with crosslinking of the HDPE matrix.

Meanwhile, when HDPE is irradiated by electron-beam, main chain scission and crosslinking are induced simultaneously with the formation of free radicals [23,24,25]. The crosslinking reaction is strongly influenced by the total absorbed dose; an increase in the absorbed dose leads to the dominance of the crosslinking reaction with the promotion of free radical formation. However, an excessive absorbed dose reverses the dominance to the main chain scission that deteriorates the mechanical properties of HDPE. Furthermore, the absorbed dose rate also affects the mechanical and chemical properties of HDPE irradiated by electron-beam [26,27,28]. The rate of free radical formation and the time for oxygen diffusion depend on the absorbed dose rate, which in turn affect the recombination of free radicals and formation of oxygen-containing species. Therefore, not only absorbed dose but also absorbed dose rate should affect the mechanical properties of irradiated HDPE-based CFRTPs.

In this work, we studied the influence of the absorbed dose rate on the mechanical properties of irradiated HDPE-based CFRTPs as a follow-up study of our previous work [20]. The absorbed dose was fixed at 400 kGy, while the absorbed dose rates were adjusted to evaluate the tensile properties and surface characteristics of the irradiated specimens. The relationship between the tensile properties and chemical structure of CFRTP and its constituents (HDPE and CF) was studied as a function of the absorbed dose rate.

## 2. Materials and Methods

### 2.1. Materials

All methods and materials are described in our previous work [20]. A brief explanation of the materials and methods is given below. Commercial long fiber thermoplastic (LFT) pellets were purchased from PlastiComp, Inc. (Winona, MN, USA). LFT pellets were composed of 30 wt.% CF (12 mm in length) that were impregnated in 70 wt.% HDPE resin. The constituents of LFT pellets, natural HDPE pellets, and CF were also purchased from PlastiComp, Inc. Information on the model and specifications of HDPE and CF were difficult to obtain from the supplier due to commercial secrecy reasons. Before specimen preparation, all materials were dried overnight at 80 °C to eliminate the moisture adsorbed on the surfaces.

### 2.2. Specimen Preparation

For tensile testing, dog-bone specimens (ASTM D638 standard) were prepared by injection molding of the dried LFT and natural HDPE pellets. Injection molding was performed using an injection molding machine (LGE-110II, LS MTRON Ltd., Anyang, Republic of Korea). The resulting specimens were named CFRTP and HDPE, respectively. These dog-bone specimens were subjected to electron-beam irradiation.

### 2.3. Electron-Beam Irradiation

The as-prepared CFRTP and HDPE dog-bone specimens and the CF tow were irradiated by an electron-beam at the Korea Institute of Industrial Technology (KITECH, Yeongcheon, Republic of Korea). The absorbed dose rate of electron-beam irradiation was adjusted to observe the changes in various properties of CFRTP, HDPE, and CF. The absorbed dose rates were varied as 3.4 kGy/s, 6.8 kGy/s, 13.5 kGy/s, and 27 kGy/s. For all irradiation experiments, the beam energy and absorbed dose were fixed at 5 MeV and 400 kGy, respectively. Therefore, samples were irradiated for 117.6 s, 58.8 s, 29.6 s, and 14.8 s at 3.4 kGy/s, 6.8 kGy/s, 13.5 kGy/s, and 27.9 kGy/s, respectively, to achieve a 400 kGy absorbed dose. All irradiation experiments were performed at air atmosphere and room temperature.

### 2.4. Tensile Testing of CFRTP and HDPE Dog-Bone Specimens

The mechanical properties of the electron-beam-irradiated CFRTP and HDPE were measured by tensile testing using a universal testing machine (UTM, INSTRON 5967, INSTRON, Norwood, MA, USA). The average and standard deviation of tensile strength (TS), strain to failure, and Young’s modulus (YM) were obtained by measuring five samples for each absorbed dose rate. The gauge length, strain rate, temperature, and humidity of all tensile tests were 50 mm, 50 mm/min, 23 °C, and 50%, respectively.

### 2.5. Tensile Testing of CF Filaments

For CF, tensile testing was conducted on an individual fiber test system (FAVIMAT+, Textechno, Mönchengladbach, Germany). The gauge length and strain rate were 25 mm and 5 mm/min, respectively. Fifteen filaments were measured for each absorbed dose rate to obtain TS and YM. The temperature and humidity of all tests were the same as tensile testing of CFRTP and HDPE.

### 2.6. Fourier Transform-Infrared (FT-IR) Analysis

FT-IR spectroscopy in attenuated total reflection was performed using an infrared microscope (IS-50, Thermo Fisher Scientific, Waltham, MA, USA). Samples were prepared by cutting them into 2 cm × 2 cm squares, and all spectra were collected by 32 scans of 4 cm^−1^ resolution.

### 2.7. X-ray Photoelectron Spectroscopy (XPS) Analysis

XPS was performed on K-Alpha (Thermo Fisher Scientific, Waltham, MA, USA) using a monochromatic Al Kα source at a 5 × 10^−7^ mbar pressure. Measurements were conducted on the fracture surface cross-section (after tensile testing) for the CFRTP and HDPE samples and the CF surface. The binding energy (BE) of C-C C1s was calibrated to 285 eV for all obtained spectra.

## 3. Results and Discussion

**Figure 1:**
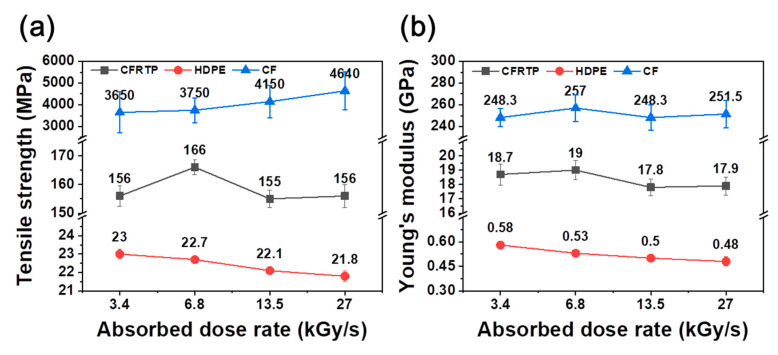
(**a**) TS and (**b**) YM of CFRTP, HDPE, and CF irradiated at various absorbed dose rates.

Figure 1 and Table 1 show the TS and YM of irradiated CFRTP, HDPE, and CF, respectively, by varying the absorbed dose rate from 3.4 kGy/s to 27 kGy/s. In the case of CFRTPs, TS increased from 157 ± 3.6 MPa to 166 ± 2.5 MPa when increasing the absorbed dose rate from 3.4 kGy/s to 6.8 kGy/s; however, TS decreased to 156 ± 4 MPa for absorbed dose rates above 13.5 kGy/s. The YM showed a minimal change, in the 17.8 ± 0.6–19 ± 0.7 GPa range, despite the absorbed dose rate variation. For HDPEs, minute levels of TS and YM gradually decreased when increasing the absorbed dose rate. Meanwhile, the TS of CF gradually increased from 3.65 ± 0.95 MPa to 4.64 ± 0.88 MPa when increasing the absorbed dose rate. The irradiated CF reached, at a 27 kGy/s absorbed dose rate, a maximum TS that was 27% higher than that of pristine CF. These CF TS incremental changes will be further investigated in a future study. The YM showed minute changes, in the 248.3 ± 8.4 GPa to 257 ± 12.3 GPa range, despite the absorbed dose rate variation. It was interesting that even though the TS of CF evidently increased with the absorbed dose rate, the TS of CFRTPs that included these CFs did not show an increase when increasing the absorbed dose rate. Therefore, various spectroscopic analyses of irradiated CFRTP, HDPE, and CF were conducted by FT-IR and XPS to elucidate the reason for these observations.

**Figure 2:**
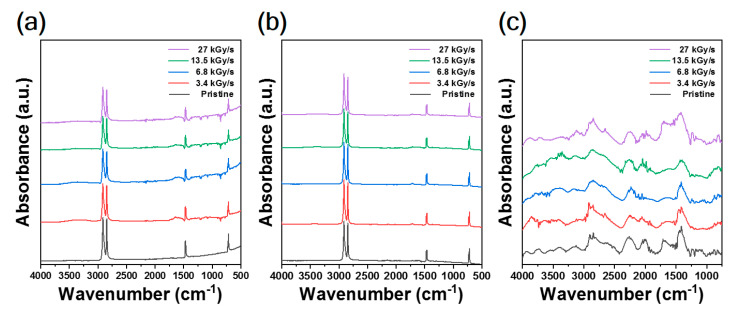
FT-IR spectra of (**a**) CFRTP, (**b**) HDPE, and (**c**) CF irradiated at various absorbed dose rates.

Figure 2 shows the FT-IR spectra of CFRTP, HDPE, and CF, respectively, irradiated at different absorbed dose rates. For pristine CFRTP and HDPE, typical vibration modes such as CH stretching at 2913 cm^−1^ and 2846 cm^−1^, CH_2_ deformation at 1471 cm^−1^ and 1455 cm^−1^, and CH_2_ rocking at 729 cm^−1^ and 717 cm^−1^, were observed, which originated from pristine HDPE [29]. After irradiation, vibration modes at 3700–3200 cm^−1^ and 1715 cm^−1^ were developed, corresponding to alcoholic (hydroxyl group) and ketone compound (carbonyl group), respectively. Additionally, the vibration mode at 1650–1550 cm^−1^ developed by irradiation that originated from the C–H bond breakage and subsequent formation of the conjugated structure in the HDPE backbone, along with unsaturated ketone compound (alkenyl group). In the case of CF, various vibration modes were observed that originated from the sizing material that the CFs were coated with. Before irradiation, vibration modes at 3200–2500 cm^−1^ and 1735 cm^−1^, presumed to be alcoholic (hydroxyl group) and ester compounds (carboalkoxy group), respectively, were observed. Additional modes at ~1685 cm^−1^, ~1620 cm^−1^, 1490–1440 cm^−1^, and 1240–1035 cm^−1^, presumed to be conjugated ketone (carbonyl group), unsaturated ketone (carbonyl group), alcoholic (hydroxyl group), and ester compounds (carboalkoxy group), respectively, were observed after irradiation.

Further analysis was conducted by comparing the IR absorbance of irradiated CFRTP, HDPE, and CF for different absorbed dose rates. In the case of HDPE, the vibration of ketone compound (carbonyl group) declined, while an alcoholic (hydroxyl group) vibration was developed by increasing the absorbed dose rate (Figure 2b). This was due to the predominant formation of hydroxyl group, rather than carbonyl group, at higher absorbed dose rates. By irradiation, the conjugated C=C structures in the HDPE backbone chain were more developed at higher dose rates. The π-electrons of unsaturated ketone compound (carbonyl group) could be delocalized to these conjugated C=C structures. As a result, the C=O double bond could be transformed into an oxygen anion (C–O^−^). Moreover, hydroxyl radicals (OH·) could be formed from air moisture and oxygen during irradiation [30,31,32]. Subsequently, the oxygen anions and hydroxyl radicals could react to form hydroxyl groups in the HDPE backbone chain [33,34,35,36]. Therefore, the increased formation of unsaturated ketone compound (alkenyl group) at a higher absorbed dose rate led to a higher tendency of hydroxyl groups to be formed compared to ketone compound (carbonyl group) in the irradiated HDPE. However, in the case of CFRTPs, the IR absorbance of the OH stretching mode was prominently decreased when increasing the absorbed dose rate, while ketone compound (carbonyl group), conjugated alkene, and unsaturated ketone compound (alkenyl group) showed minute changes despite the absorbed dose rate variation (Figure 2a). The strong and broad IR absorption centered at ~3350 cm^−1^ corresponded to intermolecular OH bonding. The reason for this opposite trend will be explained later.

**Figure 3:**
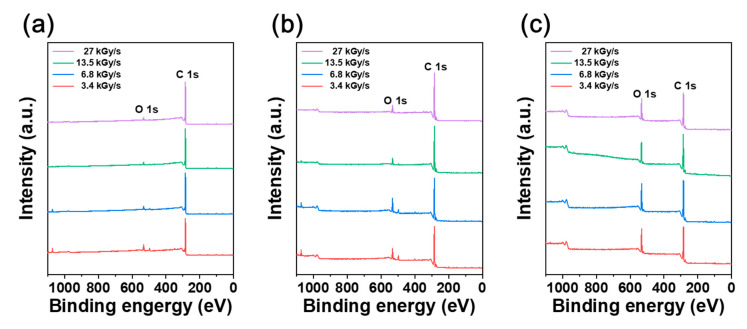
XPS survey scan spectra of (**a**) CFRTP, (**b**) HDPE, and (**c**) CF irradiated at various absorbed dose rates.

Evaluation of oxygen content and identification of oxygen functional groups were conducted by XPS analysis for different absorbed dose rates. Figure 3 displays the survey scan spectra of irradiated CFRTP, HDPE, and CF at different absorbed dose rates. All samples consisted mostly of C and O with minute amounts of Si, Na, and N, which might be impurities included in the samples. Based on these spectra, the atomic percentages and C/O ratios of each sample were evaluated and are summarized in Table 2. The oxygen contents of irradiated CFRTP and HDPE decreased for higher absorbed dose rates (Table 2). This was attributed to the shorter irradiation time for higher absorbed dose rates, which limited the diffusion time of oxygen inside the CFRTP and HDPE samples, and the subsequent reaction of oxygen with active radical species generated during irradiation. In the case of CF, the oxygen content was similar for the 400 kGy absorbed dose, despite the absorbed dose rate difference, due to sufficient oxygen supply to the CF surface during irradiation. Furthermore, it is well known that high concentrations of reactive radical species generated within a short time by a high absorbed dose rate are easy to recombine [37,38,39]. Therefore, the subsequent reaction of radical species with oxygen to form oxygen-containing chemical bonds is not suitable for higher absorbed dose rates [40,41]. The C/O ratio of CF showed minor changes, in the 4.64–5.31 at% range, while the C/O ratio of CFRTP and HDPE doubled when the absorbed dose rate increased from 3.4 kGy/s to 27 kGy/s. Based on the XPS survey scan results, the oxygen contents of irradiated CFRTP and HDPE were decreased with increases in the absorbed dose rate.

**Figure 4:**
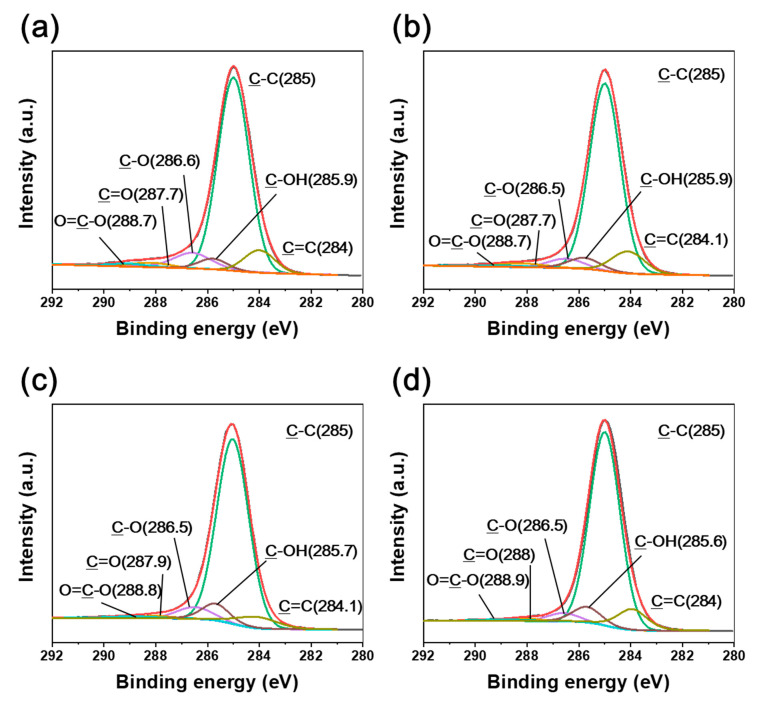
C1s XPS spectra of CFRTPs irradiated at (**a**) 3.4 kGy/s, (**b**) 6.8 kGy/s, (**c**) 13.5 kGy/s, and (**d**) 27 kGy/s absorbed dose rates.

**Figure 5:**
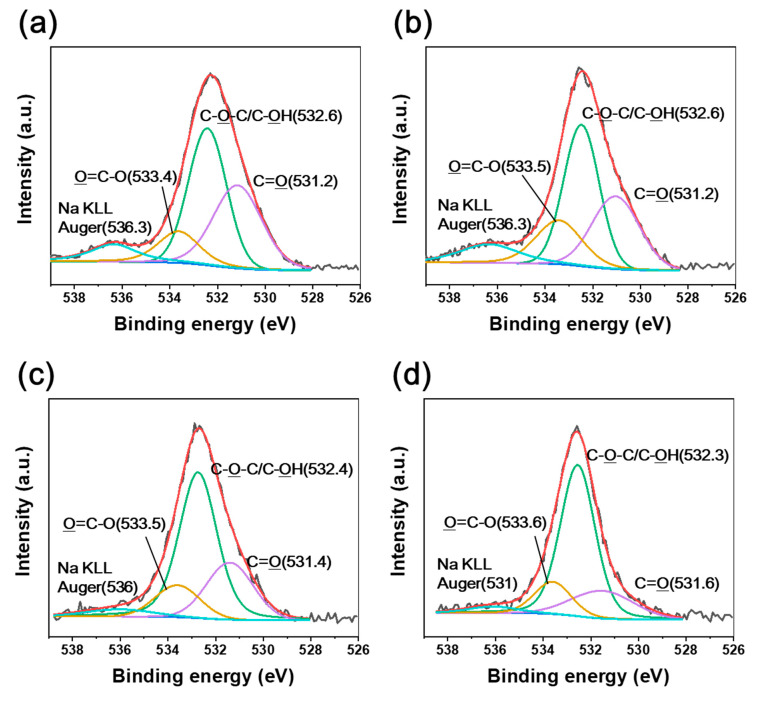
O1s XPS spectra of CFRTPs irradiated at (**a**) 3.4 kGy/s, (**b**) 6.8 kGy/s, (**c**) 13.5 kGy/s, and (**d**) 27 kGy/s absorbed dose rates.

Various oxygen-containing groups, such as C=O, O=C–O, and C–OH, were identified in the irradiated CFRTPs (Figure 4 and Figure 5). It was found that the C–OH bond developed, while C=O and O=C–O bonds declined when the absorbed dose rate increased. It is noteworthy that BE shifts of C1s and O1s were observed when changing the absorbed dose rates. The C1s and O1s binding energies of the irradiated CFRTPs for various absorbed dose rates are summarized in Table 3. In the case of the C–OH bond, a down-shift in BE for the C1s and O1s orbital electrons was observed when increasing the absorbed dose rate. However, up-shifts in the binding energies for C=O and O=C–O bonds for C1s and O1s orbital electrons were observed when increasing the absorbed dose rate. These BE shifts originated from the hydrogen bond interactions of C–OH, C=O, and O=C–O generated by irradiation at the HDPE and CF interface [42,43,44]. It is known that as the hydrogen bond interaction is enhanced, the C1s and O1s binding energies of C–OH increase because C–OH (δ^+^) acts as a hydrogen bond donor. Meanwhile, the C1s and O1s binding energies of their counterpart groups, such as C=O or O=C–O, decrease because they act as hydrogen bond acceptors (δ^−^). Therefore, it was concluded that hydrogen bond interactions between HDPE and CF in irradiated CFRTP declined when increasing the absorbed dose rate. These results were consistent with the FT-IR analysis, which showed that stronger absorption centered at ~3350 cm^−1^ corresponding to intermolecular OH bonding was observed at lower absorbed dose rates.

**Figure 6:**
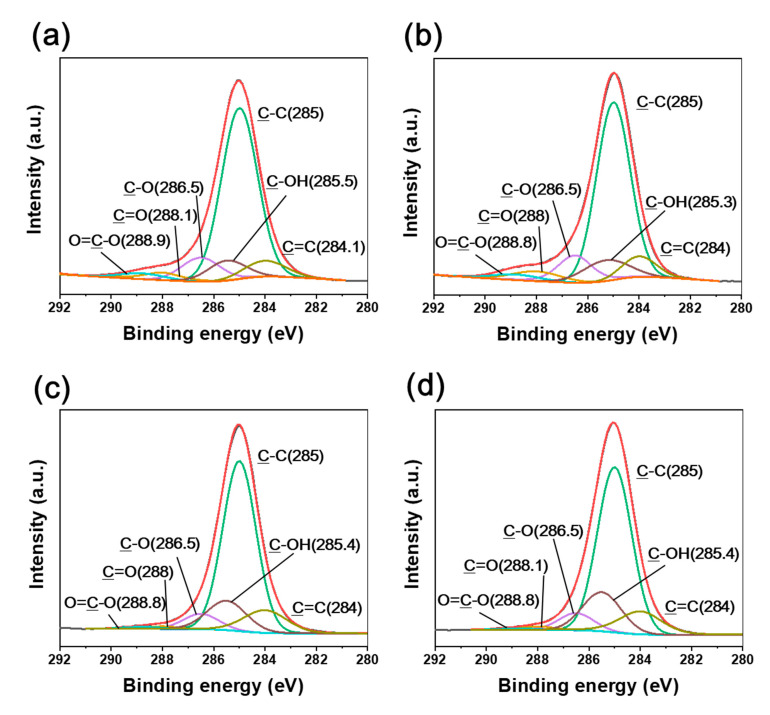
C1s XPS spectra of natural HDPE irradiated at (**a**) 3.4 kGy/s, (**b**) 6.8 kGy/s, (**c**) 13.5 kGy/s, and (**d**) 27 kGy/s absorbed dose rates.

**Figure 7:**
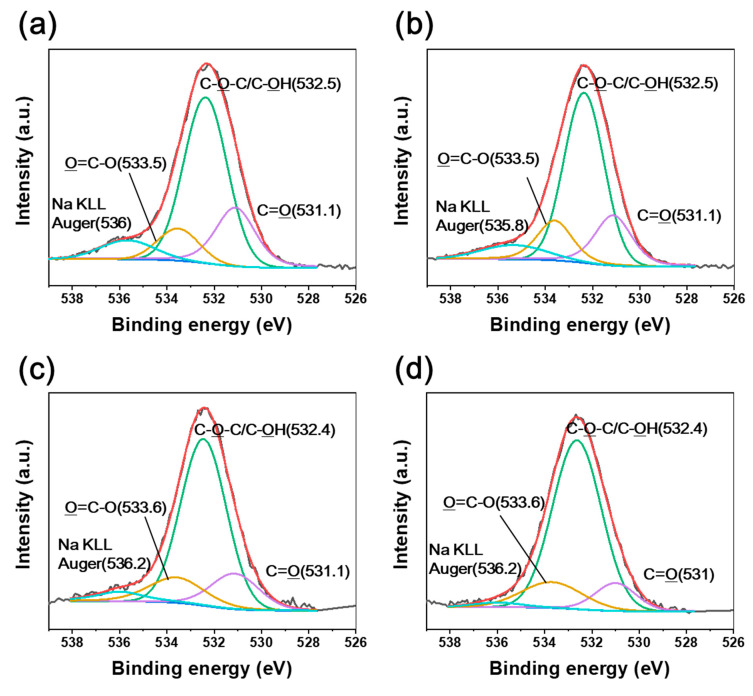
O1s XPS spectra of natural HDPE irradiated at (**a**) 3.4 kGy/s, (**b**) 6.8 kGy/s, (**c**) 13.5 kGy/s, and (**d**) 27 kGy/s absorbed dose rates.

**Figure 8:**
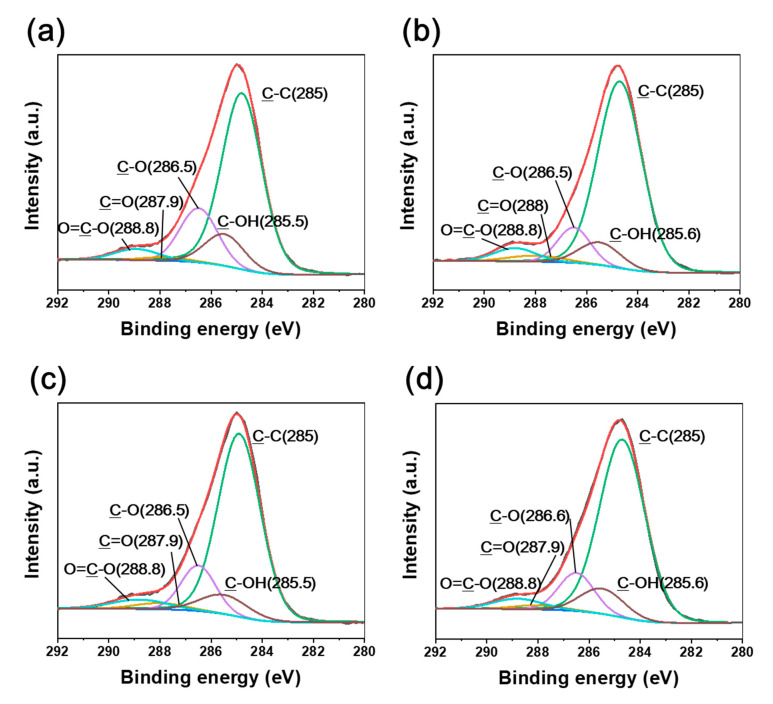
C1s XPS spectra of CF irradiated at (**a**) 3.4 kGy/s, (**b**) 6.8 kGy/s, (**c**) 13.5 kGy/s, and (**d**) 27 kGy/s absorbed dose rates.

**Figure 9:**
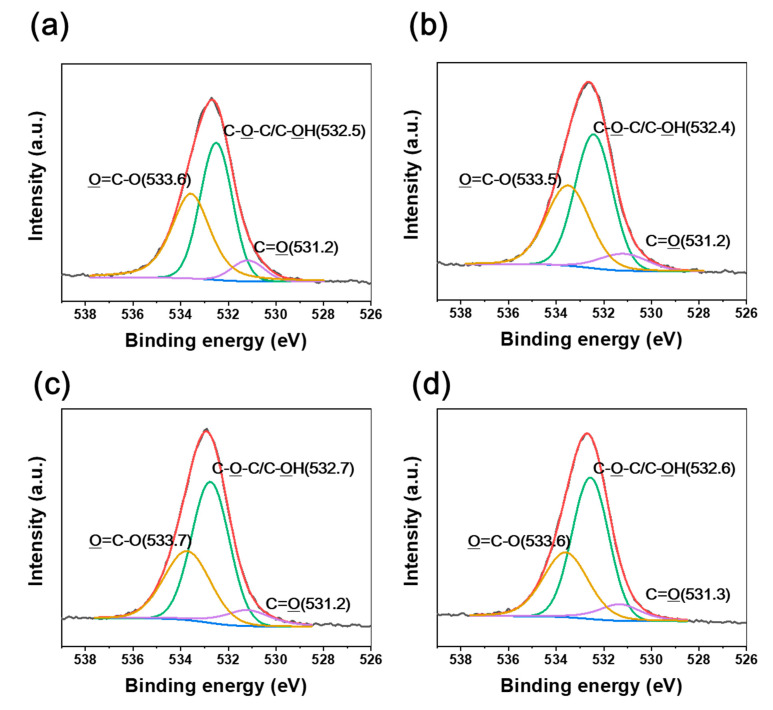
O1s XPS spectra of CF irradiated at (**a**) 3.4 kGy/s, (**b**) 6.8 kGy/s, (**c**) 13.5 kGy/s, and (**d**) 27 kGy/s absorbed dose rates.

Figure 6 and Figure 7 show the C1s and O1s spectra of natural HDPE irradiated at various absorbed dose rates. Similar to the CFRTP results, our previous study showed that chemical bonds such as C=O and O=C–O were developed by irradiation [26]. It was observed that C–OH and C–C bonds were formed, while C=O and O=C–O bonds declined when increasing the absorbed dose rate. However, negligible BE shifts were observed for irradiated natural HDPE, despite the different absorbed dose rates. Therefore, this indicated that no hydrogen bond interactions took place in natural HDPE by irradiation because of the absence of chemical bonds of the CF constituent as a counterpart. The C1s and O1s XPS spectra of the irradiated CF also showed that negligible BE shifts occurred despite the different absorbed dose rates (Figure 8 and Figure 9).

**Figure 10:**
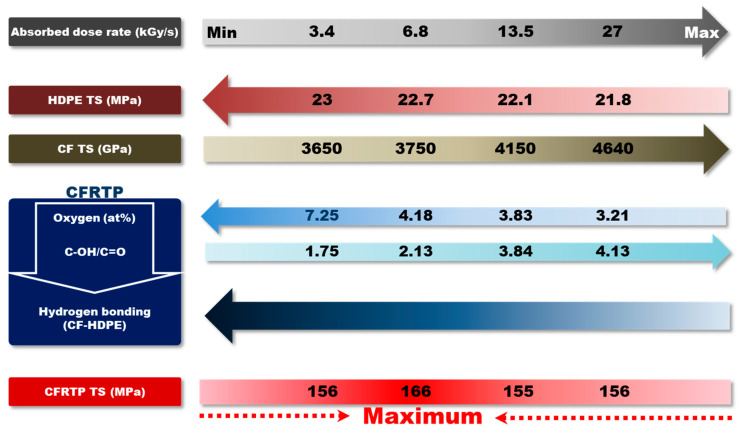
Relationships of CFRTP TS vs. HDPE TS, CF TS, and CF–HDPE hydrogen bonding as a function of absorbed dose rates.

Based on our observations, the relationships of irradiated CFRTP TS vs. HDPE TS, CF TS, and CF–HDPE hydrogen bonding as a function of the absorbed dose rates are summarized in Figure 10. In the case of CFRTP constituents, the CF TS gradually increased, which contributed to the TS increase of irradiation CFRTP until 6.8 kGy/s, while a minute decrease in HDPE TS contributed to the CFRTP TS decreasing as the absorbed dose rate increased. Meanwhile, the attractive hydrogen bonding between HDPE and CF of irradiated CFRTP decreased with the increase in the absorbed dose rate. This was attributed to the decrease in the amount of oxygen, even though the hydroxyl OH/C=O ratio increased as the absorbed dose rate was increased. This is supported by our FT-IR observation that the intermolecular bonded OH stretching, centered at ~3350 cm^−1^, gradually diminished as the absorbed dose rates increased. These results could explain the decrease in the TS of the irradiated CFRTP for absorbed dose rates higher than 6.8 kGy/s. Therefore, from the viewpoint of TS, several opposite effects occurred when increasing the absorbed dose rate of irradiated CFRTPs. The increase in CF TS was favorable, while the decline of attractive hydrogen bonding interactions between HDPE and CF was adverse for CFRTPs TS. Therefore, the irradiated CFRTP TS was maximized at an optimum absorbed dose rate of 6.8 kGy/s used in our study.

## 4. Conclusions

In this work, we studied the influence of the absorbed dose rate on the mechanical strength and fiber–matrix interaction of electron-beam-irradiated CFRTP. The electron-beam energy and absorbed dose were fixed, while the absorbed dose rates were varied as 3.4 kGy/s, 6.8 kGy/s, 13.5 kGy/s, and 27 kGy/s for all experiments. The irradiated CFRTP TS increased for absorbed dose rates of up to 6.8 kGy/s and then decreased at higher absorbed dose rates. As the absorbed dose rate increased, the CF TS gradually increased, while the HDPE TS slightly decreased. Based on the FT-IR analysis, the OH intermolecular bond was strongly developed in irradiated CFRTP at a low absorbed dose rate and gradually declined when the absorbed dose rates increased. Further XPS analysis revealed that the oxygen content of irradiated CFRTPs decreased when increasing the absorbed dose rate. Therefore, this contributed to the decrease in irradiated CFRTP TS at higher dose rates. It was noticed that although the hydroxyl OH groups developed at higher absorbed dose rates, the amount of oxygen was a more important factor for the TS of irradiated CFRTP. Finally, from the viewpoint of TS, several opposite effects occurred when increasing the absorbed dose rate of irradiated CFRTPs. The increase in CF TS was favorable, while the decline of attractive hydrogen bonding interactions between HDPE and CF was adverse for CFRTPs TS. Therefore, the irradiated CFRTP TS was maximized at an optimum absorbed dose rate of 6.8 kGy/s used in our study. Based on our results, electron-beam irradiated HDPE-based CFRTP could be applied to the products that HDPEs are currently utilized, such as hot water pipelines, gas pipelines, lightweight automobile parts, etc. 

## Figures and Tables

**Table 1:** Tensile strength and Young’s modulus of CFRTP, HDPE, and CF irradiated at various absorbed dose rates.

	Absorbed Dose Rate (kGy/s)	Tensile Strength (MPa)	Young’s Modulus (GPa)
CFRTP	3.4	156 ± 3.6	18.7 ± 0.74
	6.8	166 ± 2.5	19.0 ± 0.66
	13.5	155 ± 3.0	17.8 ± 0.60
	27	156 ± 4.0	17.9 ± 0.63
HDPE	3.4	23.0 ± 0.25	0.58 ± 0.019
	6.8	22.7 ± 0.03	0.53 ± 0.015
	13.5	22.1 ± 0.12	0.50 ± 0.015
	27	21.8 ± 0.29	0.48 ± 0.028
CF	3.4	3650 ± 950	248.3 ± 8.4
	6.8	3750 ± 580	257.0 ± 12.3
	13.5	4150 ± 740	248.3 ± 11.6
	27	4640 ± 880	251.5 ± 12.7

**Table 2:** Atomic percentage and C/O ratio of CFRTP, HDPE, and CF irradiated at various absorbed dose rates.

	Absorbed Dose Rate (kGy/s)	at%					
		C1s	O1s	Na1s	N1s	Si2p	C/O Ratio
CFRTP	3.4	89.3	7.25	1.23	1.12	1.09	12.31
	6.8	93.33	4.18	1.02	0.71	0.76	22.34
	13.5	94.38	3.83	0.68	0.63	0.47	24.62
	27	96.41	3.21	0.02	0.17	0.19	30.04
HDPE	3.4	84.51	10.28	1.7	2.2	1.3	8.22
	6.8	83.35	11.91	1.34	2.03	1.38	7
	13.5	91.22	5.77	0.66	1.52	0.82	15.82
	27	92.74	6.48	0	0.77	0	14.3
CF	3.4	78.51	16.92	–	2.01	2.56	4.64
	6.8	80.48	17.29	–	–	2.23	4.66
	13.5	80.1	15.09	–	2.31	2.5	5.31
	27	80.51	16.31	–	1.35	1.83	4.94

**Table 3:** C1s and O1s binding energies of irradiated CFRTPs for various absorbed dose rates.

Orbital		C1s			O1s		
Chemical Bond		C–OH	C=O	O=C–O	C–OH	C=O	O=C–O
Hydrogen bond interaction		Donor (δ^+^)	Acceptor (δ^−^)		Donor (δ^+^)	Acceptor (δ^−^)	
		BE					
Absorbed dose rate (kGy/s)	3.4	285.9	287.7	288.7	532.6	531.2	533.4
	6.8	285.9	287.7	288.7	532.6	531.2	533.5
	13.5	285.7	287.9	288.8	532.4	531.4	533.5
	27	285.6	288	288.9	532.3	531.6	533.6
BE shift		Down-shift	Up-shift		Down-shift	Up-shift	
